# The relationship between pulp calcifications and salivary gland calcifications

**DOI:** 10.4317/jced.51518

**Published:** 2014-12-01

**Authors:** Sumita Kaswan, Santosh Patil, Sneha Maheshwari, Farzan Rahman, Suneet Khandelwal

**Affiliations:** 1 Dept of Endodontics and Conservative Dentistry, Jodhpur Dental College General Hospital, Jodhpur (Rajasthan), India; 2 Dept of Oral Medicine and Radiology, Jodhpur Dental College General Hospital, Jodhpur (Rajasthan), India; 3 Dental Practitioner, Jodhpur (Rajasthan), India; 4 Dept of Oral andMaxillofacial Pathology, Jaipur Dental College, Jaipur (Rajasthan), India; 5 Dept of Oral and Maxillofacial Pathology, Desh Bhagat Dental College, Muktsar (Punjab), India

## Abstract

Aim: Pulp stones are discrete calcified bodies found in the dental pulp. Sialolithasis is the most common salivary gland disease. The aim of the present study was to determine the relationship between the pulp stones and salivary gland stones. Material and Methods: 196 patients were randomly selected from the out patient department for the study. The periapical radiographs for all patients were evaluated for the presence or absence of the narrowing of dental pulp chambers and pulp canals. The intra oral occlusal radiographs were also evaluated to determine the presence or absence of salivary stones. The results were compared and analyzed using the Chi-square test (p<0.001). Results: Salivary gland calcifications were detected in 5 patients. 191 patients had pulp narrowing and 118 patients had pulp stones. There was no statistical correlation between pulp narrowing and salivary stones (p>0.001) and also between pulp stones and salivary gland stones (p>0.001). Conclusions: However, the incidental findings of salivary gland stones on intra oral occlusal radiographs can provide useful information in the early diagnosis of the condition, but in the present study no significant relationship was found between the presence of pulp stones and salivary gland stones.

** Key words:**Pulp stone, salivary gland stone, periapical radiograph, occlusal radiograph.

## Introduction

The calcified structures in the dental pulps of healthy, diseased, and even unerupted teeth are known as pulp stones. They may be present in the teeth of deciduous and permanent dentition (1). These calcifications may be located in the coronal or radicular part of the pulp. Pulp stones have been described as the symptoms of the changes in the pulp tissue rather than their cause. The exact mechanism and the etiology is not known, however, various factors such as pulp degeneration, age, genetic factors, certain syndromes such as the van der Woude syndrome, epithelium rests in the pulp tissue, impaired blood supply of the pulp, periodontal disease, orthodontic treatment, operative procedures and long-standing irritants such as caries, deep fillings, or abrasion have been implicated in their formation (2,3). It has also been noted that pulpal stone formation may result when an irritated pulp, attempts to repair itself. The carious teeth of children and young adults have shown a 5 times higher incidence of pulp calcifications when compared with the non carious teeth (2). The frequency of pulp stones has been reported to increase with advancing age. A decrease in the size of the pulp chamber due to the deposition of the secondary dentin and due to the deposition of calcified masses in the root has been reported by Bernick and Nedelman (4). A few studies have reported no difference in occurrence of pulp stones between genders, while some have reported a higher prevalence of pulp stones in females (5,6). Pulp stones vary in number from 1 to 12 or more in a single tooth and the size vary from small microscopic particles to large masses occluding the pulpal space (6).

Sialolithiasis is the most common disorder of the major salivary glands. According to one of the proposed theories for its etiopathogenesis, it results from a deposition of calcium salts around a core made of desquamated epithelial cells, bacteria, mucus or foreign bodies. The submandibular salivary gland is the most common site for stone formation, given that around 60-90% of the stones are reported to be located in this gland. Approximately 10–20% of stones are found in the parotid gland and 1–5% in the sublingual gland (7,8). Around one-fourth of the cases, multiple stones have been reported (9). The size may vary from 0.1 to 30 mm. However, Drage *et al.* (10) have reported a mean size of up to 3.4 mm (range from 1.5 to 9 mm) for the parotid and submandibular stones, and a mean number of 1.67 stones (range from 1 to 5) per patient.

The pulp calcifications usually occur throughout the dentition in patients with systemic or genetic diseases like dentin dysplasia and dentinogenesis imperfecta (11). The conditions that are secondary to the calcium metabolism like hypocalcaemia, gout and renal lithiasis have been noted as the predisposing factors. A strong correlation between the chronicity of the renal disease and the pulp narrowing has been observed in the premolar and molar teeth of such patients (12). A number of studies have suggested that pulp stones results as a manifestation of systemic illnesses leading to pathological biomineralization in many parts of the body (13,14). Few others have suggested that the complex biomechanical and physiologic changes occurring in these systemic diseases do not affect the dentin and pulp, stating that no such correlation exists (15).

No previous studies have been done to establish a correlation between the presence of pulp stones and salivary stones. Thus, the present study was carried out to determine the relationship between the pulp stones and salivary gland stones.

## Material and Methods

The present study included a total of 196 randomly selected patients visiting the outpatient department of Jodhpur Dental College General Hospital to study the prevalence of salivary stones and pulp stones. Ethical clearance was obtained from the Institutional Ethical Committee. A written informed consent was obtained from the patients prior to the study. A detailed medical and dental history of all the patients was recorded. Patients with any history of cardiovascular diseases, gout, gall stones, renal stones or any other systemic diseases were excluded from the study. Patients with any attrition or abrasion, presence of radiographically observable periodontal diseases and presence of Class V restorations were also excluded from the study. A total of 2879 analogic periapical radiographs of the posterior and anterior teeth were evaluated for the presence of pulp stones. The mandibular occlusal radiographs were evaluated for the presence or absence of stones in the submandibular and sublingual glands and ducts. Since majority of the salivary stones are present in the submandibular and sublingual glands, intraoral radiographs of only these glands were examined. Also since, the study was intended to be carried out using intraoral (periapical and occlusal) radiographs only, parotid gland stones were not taken into account as these can be detected using extraoral radiographic techniques. Standard equipment (65 kVp, 15 Ma, 2.7 mm Al filt., Trophy Irix, Trophy Radiologie, Paris, France) and films (Ektaspeed, Kodak, Chalon-sur-Saone, France) were used and automatic processing (Velopex, London, UK) followed standard routines. The radiographs with poor angulations, improper exposures or faulty processing, which could lead to scoring difficulties and the radiographs with carious and restored teeth were excluded from the study. The periapical radiographs for all patients were evaluated for the presence or absence of the narrowing of dental pulp chambers and pulp canals. All the radiographs were interpreted by two examiners (oral radiologists) in a dark room by using a standard viewing box under the 2X magnification and with the peripheral light being blocked out, to ensure the accuracy of the diagnosis. Narrowing was defined as a notable reduction in the size of the pulp chamber and the pulp canals. The definite radiopaque masses inside the pulp chambers were identified as pulp stones and those inside the salivary glands were identified as salivary stones. They were scored as present or absent. The data was entered using computer software SPSS 12.0 (SPSS Inc., Chicago, USA) and analyzed using the Chi-square test. *P*-value<0.001 was considered to be statistically significant.

## Results

A total of 5965 teeth from 196 patients were evaluated. The mean age of the patients was 31.8±10.6 years. There were 102 (52%) males and 94 (48%) females. On examination of the mandibular occlusal radiographs, only 5 (2.5%) patients showed the presence of salivary stones. All the stones were present in the submandibular glands. 4 (3%) salivary stone patients and 126 (97%) normal patients showed pulp narrowing. 66 (33.6%) patients presented with no narrowing of the pulp canals ( Table 1). There was no statistically (*p*>0.001) significant relation between the presence of salivary stones and pulp narrowing. Pulp stones were observed in a total of 118 (60.2%) patients, which included 3 (2.5%) patients with salivary stones ( Table 2). There was no statistically (*p*>0.001) significant relation between the presence of salivary stones and pulp stones.

**Table 1:**
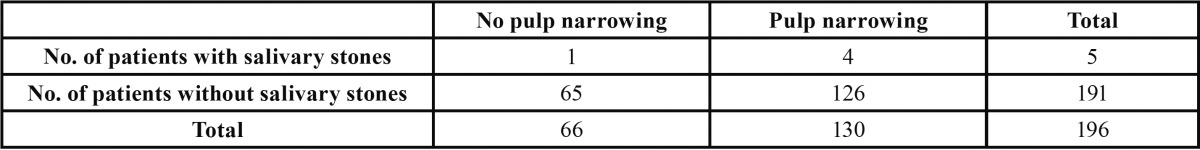
Distribution of patients with and without pulp narrowing.

**Table 2:**
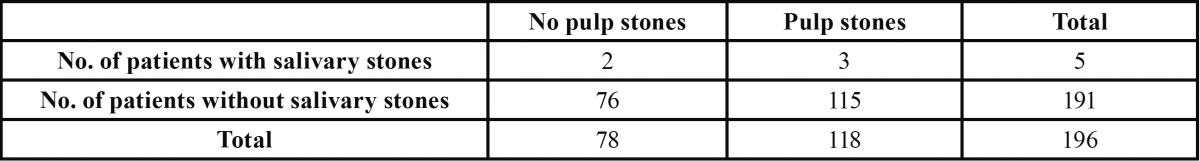
Distribution of patients with and without pulp stones.

## Discussion

Pulp stones are discrete calcifications found in the pulp chamber or pulp canals of deciduous and permanent teeth. Based on the location, pulp stones may occur freely within the pulp tissue or embedded and adherent to the dentin. The embedded stones for-med in the pulp may become enclosed within the canal walls due to the deposition of physiological dentin. They are usually located at the apical portion of the root. Odontoblasts and a calcified tissue resembling the dentine may be present on the peripheral aspect of these stones (16). The adherent pulp stones are never fully enclosed by the dentine and are less attached to the dentine. Both the types of pulp stones can cause significant obstruction of the canals and may interfere with the root canal treatment (11). The pulp stones are divided histologically into “true” or “false”(17). The true pulp stones are composed of dentin, are more irregular in shape and lined by odontoblasts. The false pulp stones are formed from the mineralization of degenerating cells of the pulp (2,16). A third type of pulp stones, ‘diffuse’ or ‘amorphous’ type is also seen in close association with the blood vessels (2).

The prevalence of pulp stones has shown wide discrepancy in various populations varying from 8-90%, depending on the study type, design, and radiographic technique employed (5,6,18,19). The various prevalence studies have been based on person and teeth numbers, while few have been based only on teeth number (17). The pulp stones are often incidental findings on radiographs and many radiological studies have investigated the incidence of pulp stones. Histological method of evaluation has been reported to yield higher values of prevalence than radiographic method. The prevalence based on radiographic examinations, has been reported to be around 20 to 25% (18). However, Hamasha and Darwazeh (18) identified a prevalence of 51.4% in Jordanian adults in a radiographic study and Ranjitkar S *et al.* (5) reported a prevalence of 46.1% in Australian adults’. The prevalence of pulp stones in the present study was reported to be 60.2%, which was higher than the above mentioned studies. 115 of the patients with pulp narrowing (126 patients) presented with pulp stones in the present study. The difference in the prevalence may be due to the variation in sample and sample size in various studies, along with the difference in the presentations of prevalence.

Sialolithiasis is the cause of 42-77% of all reported cases of salivary duct obstruction (20). Recurrent and chronic obstruction leads to saliva retention, infection and inflammation, leading to chronic salivary gland edema (7). Symptoms of sialolithiasis are similar to the symptoms of other cases of salivary duct obstruction, which include, a transient, painful, postprandial edema, which gradually retreats in 2-3 hours, and pain during meals. The saliva production also decreases (21). The prevalence of salivary stones in the general population has been estimated to be about 1.2% according to post mortem studies, but the prevalence of salivary stones that cause symptoms in the general population is estimated to be about 0.45% (22). The prevalence of salivary gland stones in the present study was found to be 2.5%, which was quite higher than that mentioned in the literature.

Sialolithiasis is most commonly seen in the submandibular gland since; it produces a particularly viscous, mucous and more alkaline saliva, which has a relatively high concentration of hydroxyapatites and phosphates. The opening of the Wharton’s duct is also narrower than the diameter of the whole duct. Furthermore, the duct ascends towards its opening, which also leads to the retention of saliva. All these factors predispose to the increased occurrence of calculi in the submandibular glands (21,23). Keeping this in mind, and also the fact that extraoral radiographs were not intended to be done, the present study included examination for only submandibular and sublingual glands, which could be evaluated using single intraoral occlusal radiograph. All the salivary stones (5 patients) detected in the present study were located in the submandibular gland itself. 85% of the submandibular gland stones are located in the Wharton’s duct, and the rest 15% in the gland parenchyma, which do not tend to cause significant clinical symptoms, unlike the stones in the Wharton’s duct which produce symptoms of inflammation or pain (23). The proximal segment of the Wharton’s duct, where the duct wraps around the posterior edge of the mylohyoid muscle, at a steep angle is the most common site for calculi formation. Sjögren’s syndrome and sarcoidosis also promote the formation of sialoliths (21).

No previous studies have been done till date to study the correlation between the presence of pulp stones and salivary stones. Hence, the present study was designed to find if any such relation exists. A total of 130 patients showed pulp narrowing, of which 4 patients had salivary gland stones. However, only 3 of the patients with salivary stones had pulp stones and 115 of the normal patients had pulp stones. The presence of pulp calcifications along with the stones in the salivary glands however showed no statistically significant relationship (*p*>0.001). The intraoral radiographs such as bitewing and periapical radiographs are the only means of examining pulp stones non-invasively in such studies. These radiographs when compared for their efficacy in the diagnosis of pulp stones showed no significant difference, and hence periapical radiographs were used in the present study to determine pulp stones.

The present study however, did not report any significant association between pulp stones and salivary gland stones but it seems to be of significant clinical importance. The intraoral radiographs are easy screening methods that frequently reveal the presence of calcified structures in the pulp, but these may not be conducive to provide any information about salivary gland stones. Further large scale, multi-institutional studies are encouraged to establish any positive correlation between pulp stones and salivary gland stones. It is thus suggested that careful evaluation of routine dental radiographs could serve as a significant prognostic tool for early identification of salivary gland calculi.
